# Antiepithelial-Mesenchymal Transition of Herbal Active Substance in Tumor Cells via Different Signaling

**DOI:** 10.1155/2020/9253745

**Published:** 2020-04-17

**Authors:** Xiaoji Cui, Qinlu Lin, Ping Huang, Ying Liang

**Affiliations:** Molecular Nutrition Branch, National Engineering Laboratory for Rice and Byproduct Deep Processing, College of Food Science and Engineering, Central South University of Forestry and Technology, Changsha, 410004 Hunan, China

## Abstract

Epithelial-mesenchymal transition (EMT) is a biological process through which epithelial cells differentiate into mesenchymal cells. EMT plays an important role in embryonic development and wound healing; however, EMT also contributes to some pathological processes, such as tumor metastasis and fibrosis. EMT mechanisms, including gene mutation and transcription factor regulation, are complicated and not yet well understood. In this review, we introduce some herbal active substances that exert antitumor activity through inhibiting EMT that is induced by hypoxia, high blood glucose level, lipopolysaccharide, or other factors.

## 1. Introduction

Epithelial cells differ from mesenchymal cells in phenotype and function. Epithelial cells have apicobasal polarity characteristics and are tightly connected to each other through tight junctions, gap junctions, and adherens junctions [1]. Epithelial cells form layers on cavities, blood vessel surfaces, and organs throughout the body. In contrast, mesenchymal cells lack polarization and are spindle shaped, which allows them to interact with each other only through focal points [2]. Epithelial-mesenchymal transition (EMT) is defined as a biological and pathological process through which epithelial cells differentiate into mesenchymal cells. During EMT, epithelial cells forfeit their characteristics (such as cellular polarity, pseudopodia formation, and disintegration of the E-cadherin-related cell-cell adhesion) and begin to display properties of stromal cells (such as higher activity, mobility, elongated and spindle shaped, and fibroblastoid aspect). As a physiological process, EMT is involved in embryonic development including mesoderm and neural tube formation and wound healing procedures. As a pathological process, EMT contributes to organ fibrosis, tumor invasion, and metastasis in cancer progression [3–5].

Herbal active substances contain natural active ingredients that are extracted from plants and are closely related to human health. For instance, Yue et al. found that 5 *μ*mol/L lycopene could protect cardiomyocytes against oxidative damage of mtDNA induced by ischemia/reperfusion (I/R) injury [6]. Kim et al. found that after treating with 20 *μ*mol/L baicalin, dendritic cells (DCs) weaken the T helper type 1 immune response induction and normal cell-mediated immune response [7]. Applying ovalbumin- (OVA-) sensitized mouse models, Sato et al. [8] found that dietary supplementation of *α*-carotene and *β*-carotene (approximately 300 *μ*g/kg) can inhibit food allergic reactions in mice. None of them revealed any pollutants and side effects. Furthermore, these substances are not only extensive resources but are also able to reduce the psychological fear caused by chemical drugs' side effects because of the green features in their properties. In line with people's pursuit of what is “purely natural,” it is not surprising that they are easily accepted. Some herbal active substances are introduced here.

Compared to tocopherols, tocotrienols are predominantly absent in fruits and vegetables but abundant in palm oil, rice bran oil, and barley oil, and they provide excellent physiological antioxidant, cholesterol-reducing, and anticancer activities [9]. Compared to biochanin A, isoflavone, isolated from red clover, cabbage, or alfalfa, has an anti-inflammatory effect on lipopolysaccharide- (LPS-) induced inflammation in human umbilical vein endothelial cells (HUVEC). Specifically, isoflavone can activate peroxisome proliferator-activated receptor *γ* (PPAR-*γ*), thereby attenuating nuclear factor kappa-light-chain-enhancer of activated B (NF-*κ*B) activation and lipopolysaccharide- (LPS-) induced inflammation [10]. *β*-Sitosterol presents antidiabetic as well as antioxidant effects. Ming et al. showed that following *β*-sitosterol treatment, the levels of glycated hemoglobin, serum glucose, and nitric oxide were significantly decreased in streptozotocin-induced diabetic rats [11]. Naturally, the active products, such as those mentioned here, could be further divided into bioactive peptides, alkaloids, flavonoids, and saponins, which are widely applied in the pharmaceutical, agricultural, and food industries.

In this review, we discuss EMT classification, physiological and pathological function, mechanism, and signaling pathways. Furthermore, we present some plant bioactive substances extracted from Chinese herbs that have anti-EMT activity.

## 2. EMT Classification

EMT is divided into three biological contexts: developmental (Type I), fibrosis and wound healing (Type II), and cancer (Type III) [12]. Type I EMT is associated with embryonic development and organ formation. In the early stages of embryogenesis, EMT is necessary for embryo formation and placenta formation. Thus, trophectoderm cells enable the invasion of the endometrium and appropriate placenta placement through EMT, allowing nutrient and gas exchange in the embryo. In late embryogenesis and gastrulation, EMT is involved in creating the primitive streak in amniotes and the ventral furrow in *Drosophila* [13]. Compared to other EMT types, mesenchymal cells differentiated from the epithelium still have the potential to generate secondary epithelia, the so-called mesenchymal-epithelial transition (MET) [14, 15]. Mesenchymal cells, in turn, also make up many epithelial mesodermal organs from the primitive streak (such as notochord and somites) through MET [16].

Type II EMT contributes to tissue regeneration, wound healing, and fibrosis. During wound healing, the keratinocytes near the wound undergo EMT, become fibroblasts, and migrate to repair the damaged tissues [17]. Similarly, a reepithelialization, or MET, occurs once the wound is healed [18].

Type III EMT is related to metastasis of malignant tumors. In early stages, malignant cells invade the local tissue as a result of EMT. Primary tumor cells inhibit E-cadherin expression leading to a breakdown of cell-cell adhesion, a breach in the basement membrane of the vessels, and invasion into the bloodstream. Conversely, circulating tumor cells spread into several organs through the bloodstream to form colonies through clonal outgrowth mediated by MET at these metastatic sites. Therefore, EMT and MET contribute to the initiation and completion of the invasion-metastasis cascade, respectively [19, 20].

## 3. EMT Mechanisms

**Figure 1:**
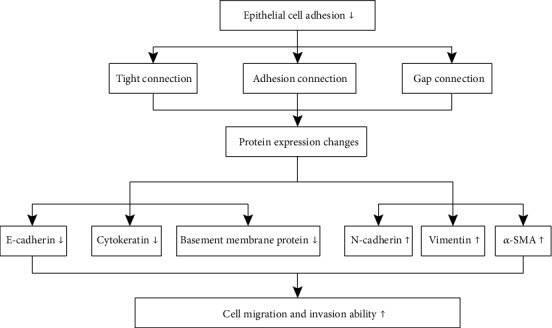
Mechanism of EMT-mediated tumor cell metastasis. Pathological factors impair epithelial cell adhesion ability resulting in the loss of tightly connected epithelial cells, a decrease in adhesive junctions, and the opening of gap junctions. Consequently, a large number of tumor metastasis-related proteins (including E-cadherin, cytokeratin, basement membrane protein, N-cadherin, vimentin, and *α*-SMA) are regulated to improve the ability of tumor cell migration and invasion.

A calcium-dependent cell-cell adhesion glycoprotein, cadherin (E-cadherin), is the most important tight junction structure in the epithelium. Impaired E-cadherin tight junctions are likely an underlying essential mechanism of EMT [21]. The E-cadherin gene (CDH1) is located on human chromosome 16q22 [22], of which the encoded protein has a calcium-binding site that promotes protein adherence to form tight cell-cell connections. Mutations or deletions in the E-cadherin protein alter the calcium-binding sites resulting in damage to the cell-cell adhesion structure and altered protein-catenin binding, which changes the cell cytoskeleton [23]. EMT-mediated mutations of epithelial cells decrease cell-cell adhesion and increase cell separation and migration. The regulation pathways of EMT-mediated tumor cell metastasis are summarized in Figure 1.

Transcription factor regulation plays an important role in EMT. Most transcription factors can activate EMT processes through directly or indirectly repressing E-cadherin. Therefore, transcription factors that suppress E-cadherin are considered EMT-transcription inhibitors. Zinc finger protein SNAI1 (SNAIL), SNAI2 (Slug), zinc finger E-box-binding homeobox 1 (ZEB1), ZEB2, Kruppel like factor 8 (KLF8), and enhancer binding factors 47 (E47) competitively bind to enhancer box (E-box) or boxes II and III of the pea rbcS 3A gene (GT boxes) consensus sequences on the E-cadherin promoter region to repress E-cadherin transcription. In contrast, transcription factors, such as twist-related protein (TWIST), can upregulate the mesenchymal cell marker protein N-cadherin to promote EMT [24]. A number of studies showed that cancer cells can trigger TWIST to inhibit E-cadherin expression and stimulate N-cadherin expression [25, 26]. Meanwhile, TWIST is reported to activate protein SET 8 (SET8) to induce histone H4K20-mediated demethylation [27]. In addition, SNAIL, TWIST, and ZEB increase the expression of mesenchymal phenotype markers (including vimentin, fibronectin, and N-cadherin) to upregulate matrix metalloproteinases (MMPs) resulting in tumor cell EMT and metastasis [28].

Transforming growth factor-*β* (TGF-*β*) signaling pathways are involved in all three types of EMT [29, 30]; TGF-*β* regulates cellular morphology, proliferation, differentiation, and apoptosis. In early stages of cancer, TGF-*β* inhibits epithelial cell proliferation; however, in the late stages, TGF-*β* promotes tumor cell invasion and migration. Binding of TGF-*β* to its receptor activates Smad2 and Smad3 signaling pathways through growth differentiation factor 15 (GDF15) [31] which inhibit the transcriptional activity of E-cadherin, thereby promoting EMT [32]. TGF-*β* upregulates SNAIL and ZEB expression to regulate EMT in heart development, palatogenesis, and cancer [33]. It was reported that the Wnt pathway triggers EMT in gastrulation, cardiac valve formation, and cancer [34]. *β*-Catenin is an essential molecule in the Wnt signaling pathway. Activation of the Wnt pathway results in the translocation of *β*-catenin into the nucleus where it activates transcription factor (TCF)/lymphoid enhancer binding factor (LEF) and depresses transcription of Wnt target genes (Axin2, CyclinD1, and Myc), thereby inducing EMT [35]. Wnt signaling promotes expression of SNAIL and the mesenchymal marker, vimentin, in breast cancer cells [36]. Other signaling pathways, including mitogen-activated protein kinase (MAPK) [37], Notch [38], fibroblast growth factor (EGF) [39], and hepatocyte growth factor (HGF) [40], are reported to participate in EMT. In addition, some biomolecules can target key factors that regulate the EMT signaling pathway to cause pathological changes in cancer cells. For example, in colorectal cancer, miR-146a polymorphism is related to liver metastasis [41]. The hypoxia-, high blood glucose-, and lipopolysaccharide- (LPS-)/transforming growth factor- (TGF-) induced EMT will be introduced later.

## 4. Anti-EMT Activity of Botanical Active Substances

### 4.1. Hypoxia Signaling

During the development and progression of a tumor, hypoxia is a common feature in the tumor microenvironment. Under hypoxic conditions, the phenotype, biological behavior, and metabolic mode of tumor cells are changed, which directly alters the tumor cells formation and resistance, thereby promoting tumor invasion and metastasis [42]. HIF-1 is a dimer consisting of *α* and *β*subunits. HIF-1*α* is a transcription factor involved in cellular oxygen-signaling regulation. The transcriptional activity of HIF-1*α* is regulated by O_2_. In normal oxygen conditions, HIF-1*α* is degraded by a tumor suppressor, the von Hippel-Lindau suppressor. Under hypoxic conditions, however, HIF-1*α* translocates into nuclei to induce HIF-1*α* expression. In the nucleus, HIF-1*α* binds to HIF-1*β* to form a stable and active HIF-1, which then combines with DNA on HRE (hypoxic response element), together constituting a transcriptional initiation complex that triggers hypoxia-related gene transcription, leading to a series of cell hypoxia adaptive responses. The C-terminal transcriptional activation region (TAD-C) of HIF-1*α* also interacts with the coactivator CBP/p300 to promote transcription in hypoxic conditions [43]. On the other hand, HIF can promote the migration and invasion of tumor cells through regulating the related transcription factors (Snail, Slug, Twist, ZEB1, SIP1, etc.). Under hypoxic conditions, the levels of Snail and HIF-1 are upregulated in tumor cells, which decrease E-cadherin expression. Furthermore, hypoxia may directly increase the level of Snail and promote EMT; in addition, the HIF-1*α* target gene LOX, uPA, and related genes facilitate the Snail level to lead to EMT, suggesting that hypoxia can also indirectly adjust the Snail level [44]. The hypoxic microenvironment increases expression of HIF-1*α* to promote EMT in hepatoma cells and facilitate their migration and invasion [45, 46]. Studies about microRNA found that miR-219 would be downregulated under hypoxic conditions and upregulated by the suppression of HIF-1*α*. Moreover, miR-219 can affect the proliferation and migration of hepatocellular carcinoma (HCC) cells under hypoxia [47].

Some botanical active substances inhibit hypoxia-induced tumor cell growth by suppressing EMT. Curcumin, extracted from the turmeric rhizome of ginger, has anti-inflammatory and anticancer activities [48]. Duan et al. [49] investigated the mechanism of curcumin's anticancer activity and found that HepG2 cells treated with 10 *μ*mol/L curcumin had significantly reduced HIF-1*α* protein levels compared with the control group, and these levels decreased with the increase of curcumin concentration; thus, curcumin inhibits HIF-1*α* expression and decreases EMT in human hepatoma HepG2 cells. Chang et al. [50] found that 10 *μ*mol/L curcumin induced the inactivation of HIF-1*α* in HepG2 cells, which prohibited EMT transduction, tumor proliferation, and migration through the activation of HIF-1*α* downstream pathway.

**Figure 2:**
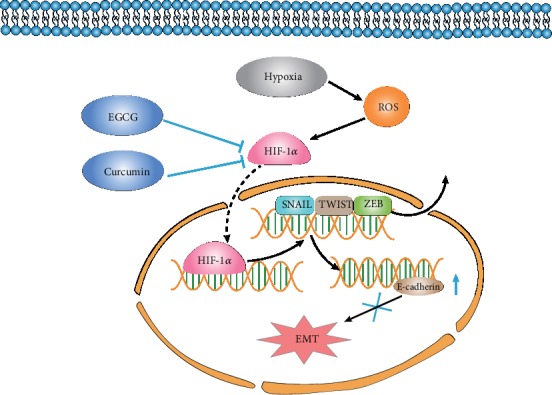
Botanical active substances prevent hypoxia-induced EMT. Botanical active substances, such as curcumin and EGCG, prevent hypoxia-induced EMT through the inhibition of HIF-1*α* and HIF-I*α*-triggered gene (including SNAIL, TWIST, and ZEB) expression.

Epigallocatechin gallate (EGCG) (also known as epigallocatechin-3-gallate) is the ester of epigallocatechin and gallic acid and is derived from unique tea catechins. Huang et al. [51] found that 40 *μ*M EGCG phosphorylates protein kinase C *δ* (PKC*δ*), which activates acid sphingomyelinase (ASM) to induce chronic myeloid leukemia (CML) cell death. In addition, this pathway can be inhibited by the soluble guanylate cyclase inhibitor NS2028. These results suggest that the anticancer property of EGCG is through the cGMP/ASM pathway in CML cells. Furthermore, Wang et al. [52] found that green tea polyphenols in EGCG downregulate HIF-1*α* expression, resulting in increased expression of miR-200, decreased EMT, and decreased invasion and migration of lung cancer cells. In another study, Shi et al. [53] verified that EGCG attenuates HIF-1*α*, vascular endothelial growth factor (VEGF), cytochrome c oxidase polypeptide II (COX-2), phosphorylated-RAC-alpha serine/threonine-protein kinase (p-Akt), phosphorylated-extracellular signal-regulated kinases (p-ERK), and vimentin protein levels in nicotine-activated A549 (lung cancer) cells. These alterations significantly inhibit nicotine-induced angiogenesis, migration, and invasion (EMT). Hypoxia induced ROS and upregulation of the expression of hypoxia-inducible factor, thus accelerating the tumor cell EMT. The active substances from plant sources have an inhibitory effect on tumor cell EMT induced by hypoxia and inhibition and downregulation of HIF-1 expression (see Figure 2).

### 4.2. TGF-*β* Signaling

Transforming growth factor-beta (TGF-*β*) is a crucial factor to regulate tumor EMT. A large number of literature has reported the molecular mechanism of TGF-*β*-mediated EMT, which is mainly divided into Smad-dependent [54] and Smad-independent pathways [55]. TGF-*β* combines with the TGF-*β* receptor II (TGFBR2) on the cell membrane to form the TGF-*β*/TGFBR2 complex. After TGFBR2 phosphorylation, the TGF-*β*/TGFBR2 complex then activates/phosphorylates the TGF-*β* receptor I (TGFBR1) to upregulate the downstream gene, Smad2/3. Smad2/3 can combine with Smad4 and translocate into the nucleus to associate with a corresponding DNA binding site to trigger gene transcription (such as Snail and ZEB). During the development of diabetic nephropathy, renal tissue TGF-*β*1 expression is significantly upregulated, which activates the Smad signaling pathway and induces tubular epithelial cell EMT and renal fibrosis. Thus, high glucose and renal tissue TGF-*β*1/Smad/Snail signaling can induce EMT resulting in renal fibrosis [56]. The Smad-independent pathway reveals an important role in the TGF-*β*-induced EMT process as well. PI3K/Akt kinase inhibitors significantly prevent the TGF-induced alterations in the cell morphology and downregulation of E-cadherin expression [57], indicating that the PI3K/Akt signaling pathway is activated by TGF-*β*, which is involved in TGF-*β*-mediated EMT through the Smad-independent mechanism. Recent studies have shown that miR-140-5p is a direct target of the oncogene Flap Endonuclease 1 (FEN1). MiR-140-5p can partially eliminate the overexpression of FEN1 to inhibit TGF-*β*1-mediated EMT in HCC cells [58].

There is a higher content of ferulic acid in wheat bran and rice bran. Wei et al. [59] found that 50 *μ*mol/L ferulic acid significantly inhibited TGF-*β*1-activated Smad2/3 signaling and TGF-*β*1-induced Snail, N-cadherin, and E-cadherin protein expression. These effects result in the suppression of lung cancer cell invasion and migration in vitro. Nobiletin, the main ingredient of citrus peel, has been found to attenuate the H1299 (human lung carcinoma cell line) cells' invasion and migration and downregulate the expression of EMT-related transcription factors Twist, Snail, and ZEB1/2, thereby inhibiting the EMT process of lung cancer cells [60]. In addition, Nobiletin could regulate the EMT process by increasing the expression of Snail, Slug, Twist, and ZEB1 to prohibit TGF-*β*-induced Smad transcription activity, therefore inhibiting the growth of lung metastatic nodules in nude mice [61]. Anthocyanin is reported to alter the TGF-*β*-induced fibronectin and Snail expression to reduce the invasive ability of human U-87 glioblastoma and prevent the migration of pancreatic cancer cells through Smad2 dephosphorylation and EMT inhibition in a dose-dependent manner [62].

**Figure 3:**
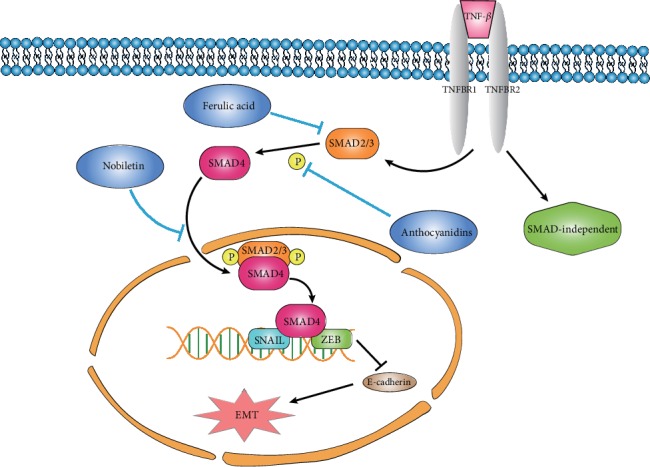
Botanical active substances prohibit Smads/Snail signaling of EMT. Botanical active substances (including anthocyanidins, ferulic acid, and nobiletin) can exert their anticancer activity through the inhibition of TGF-*β*/Smads/Snail signaling to prohibit the EMT process.

Tumor cells are susceptible to TGF-*β*1 activation through Smads/Snail signaling to induce tumor cell EMT to aggravate cellular inflammation, proliferation, and migration, therefore promoting tumor cell deterioration. These plant-derived active substances exert their anticancer activity through inhibiting Smads/Snail signaling to block EMT (Figure 3).

### 4.3. Ras/Mitogen-Activated Protein Kinase (MAPK) Signaling

MAPK signaling pathway is an important non-Smad-dependent signaling pathway, which is commonly found in mammals and involves many signal pathways in the cell to regulate cellular proliferation, differentiation, transformation, and apoptosis. The MAPK family includes three types of phosphokinases: extracellular signal-regulated kinases (ERK), c-Jun N-terminal kinases (JNK), and P38 MAPK. ERK is significantly activated during the TGF-*β*-induced EMT process, which directly increases the invasive ability of cancer cells [63]. In addition, JNK has been shown to be involved in the cancer cell phenotype differentiation and TGF-*β* can activate the p38 MAPK signaling pathway [64]. During the EMT process of HCC cells, it was found that when HCC cells were treated with the miR-140-3p mimic or siRNA-GRN, GRN (granulin) expression would be downregulated and the expression of the MAPK signaling pathway-related genes also decreased, like N-cadherin and vimentin; however, the expression of E-cadherin is exactly the opposite. GRN silencing by inhibiting miR-140-3p can reverse the activation of the MAPK signaling pathway which would induce EMT. This shows that miR-140-3p can confer suppression of the MAPK signaling pathway by targeting GRN, thus inhibiting EMT, invasion, and metastasis in HCC [65].

**Figure 4:**
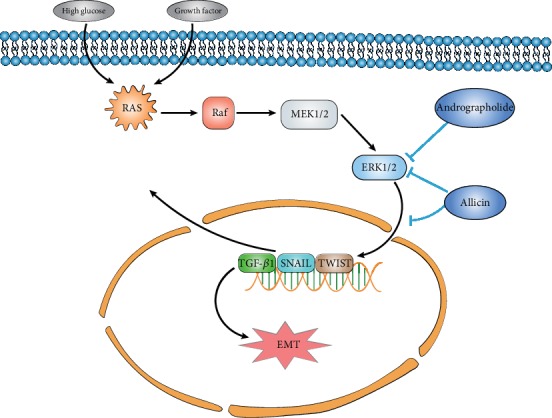
Botanical active substances restrain the EMT process through ERK1/2 signaling. High glucose or some growth factors can induce cancer cell EMT through the ERK1/2 signaling pathway. Botanical active substances, such as andrographolide and allicin, can imprison the EMT process through the dephosphorylation (inactivation) of ERK1/2.

Andrographolide is a kind of diterpene lactone compound extracted from the *Andrographis paniculata* plant, which is the main active ingredient of *Andrographis paniculata* and reveals many physiological effects, such as anti-inflammatory, antifibrosis, and antitumor [66]. Johar et al. [67] showed that during the TGF-*β*2- and bFGF-induced EMT process, andrographolide treatment significantly improved the epithelial marker pax6 and E-cadherin protein expression levels and reduced the EMT marker *α*-SMA. Connexins and collagen IV expression are regulated by dephosphorylation of ERK and JNK. High glucose can induce EMT in HK-2 cells. Allicin treatment reverses high glucose-induced upregulation of *α*-SMA, vimentin, and collagen I; increases the expression of E-cadherin and TGF-*β*1; and significantly downregulates p-ERK1/2 in a dose-dependent manner. These results suggest that allicin restrains the EMT process through the regulation of the ERK1/2-TGF-*β* signaling pathway. The ERK1/2 signaling pathway is easily activated based on external stimulation (such as high glucose or some growth factors). Botanical active substances, either andrographolide or allicin, restrain the EMT process through the dephosphorylation of ERK1/2 (Figure 4).

### 4.4. Wnt Signaling

**Figure 5:**
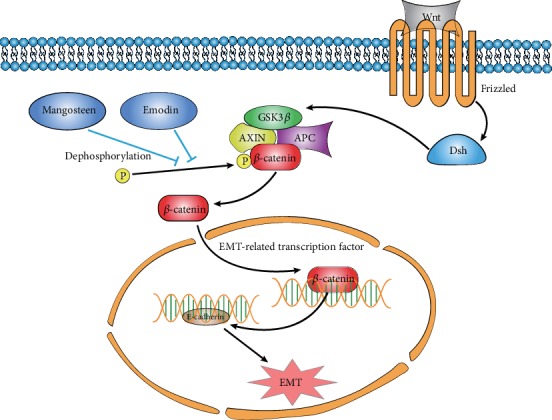
Botanical active substances restrain the EMT process through Wnt signaling. Botanical active substances, such as emodin and mangosteen, can prohibit the Wnt signaling through dephosphorylation (inactivation) of the key factor, *β*-catenin, to inhibit the cancer cell EMT process.

Wnt signaling pathways consist of the canonical Wnt pathway, the noncanonical planar cell polarity pathway, and the noncanonical Wnt/calcium pathway [68]. The key protein *β*-catenin of the Wnt signaling pathway mainly mediates cell growth and migration-related signals and mediates tumor invasion and metastasis. Activation of the Wnt signal can increase the expression of *β*-catenin, thereby downregulating the expression of E-cadherin and upregulating vimentin, both of which are marker factors of the downstream of epithelium EMT [69]. Wnt is aberrantly activated in tumors; the association of Wnt protein with Fz curl receptor in the cellular membrane activates the loose protein (dishevelled (Dsh)) to inhibit GSK3*β*, resulting in the dephosphorylation and degradation of *β*-catenin, which then translocates into the nucleus to trigger EMT-related gene transcription (Figure 5). *Panax notoginseng* (PN) targets the Wnt/*β*-catenin signaling pathway in rats. By increasing the expression of Wnt1, *β*-catenin, and Snail, PN can upregulate the expression of desmin and *α*-SMA protein and reduce the expression of nephrin, thereby ameliorating podocyte EMT [70]. Emodin is extracted from *Polygonum cuspidatum*, rhubarb, and other rhizomes from a class of bioactive substances which possess anti-inflammatory, antioxidative, and other pharmacological activities. Studies have shown that emodin can weaken epithelial ovarian cancer cell invasion and metastasis through the inhibition of the EMT process. After treatment with emodin, the expression of *β*-catenin and ZEB factor was significantly decreased in a concentration-dependent manner [71]. Ahmad et al.'s study found that mangosteen can reduce the expression of *β*-catenin through the Wnt signaling pathway to prevent the breast cancer cell EMT process in mice experiments [72].

## 5. Conclusion

Several plant-derived active substances execute their anticancer activity through EMT regulation. At the molecular level, these substances reduce expression of the epithelial markers E-cadherin, HIF-1*α*, and/or TGF-*β*1 and increase expression of the mesenchymal markers vimentin and N-cadherin, which inhibit cancer cell migration and invasion, therefore downregulating the EMT process. Numerous studies demonstrate that many signaling pathways are involved in the EMT process during tumor cell growth, including Wnt, TGF-*β*, and MAPK signaling pathways [73, 74]. However, most studies on plant-derived active substances and tumor cell EMT regulation did not provide detailed mechanisms. Future studies should give more attention to the investigation of the Wnt, MAPK, Notch, FGF, and HGF signaling pathways. Furthermore, most current studies that investigated the activities of plant bioactive substances on EMT regulation were performed in vitro only. Future research should explore in cancer animal models in vivo, which would provide more valuable data about anticancer/EMT mechanisms. Mechanistic studies of the botanical active substance's activities on tumor EMT inhibition will provide theoretical evidence to support the development of botanical active substances for new cancer therapies with fewer side effects.
